# Strength of Dry and Wet Quartz in the Low‐Temperature Plasticity Regime: Insights From Nanoindentation

**DOI:** 10.1029/2021GL094633

**Published:** 2022-01-27

**Authors:** Alberto Ceccato, Luca Menegon, Lars N. Hansen

**Affiliations:** ^1^ Dipartimento di Scienze Biologiche, Geologiche ed Ambientali—BiGeA Università di Bologna—Alma Mater Studiorum Bologna Italy; ^2^ Department of Geosciences The Njord Centre University of Oslo Oslo Norway; ^3^ Department of Earth Sciences University of Oxford Oxford UK; ^4^ Department of Earth and Environmental Sciences University of Minnesota Minneapolis MN USA

**Keywords:** nanoindentation, low‐temperature plasticity, quartz, dislocation glide, hydrolytic weakening

## Abstract

At low‐temperature and high‐stress conditions, quartz deformation is controlled by the kinetics of dislocation glide, that is, low‐temperature plasticity (LTP). To investigate the relationship between intracrystalline H_2_O content and the yield strength of quartz LTP, we have integrated spherical and Berkovich nanoindentation tests at room temperature on natural quartz with electron backscatter diffraction and secondary‐ion mass spectrometry measurements of intracrystalline H_2_O content. Dry (<20 wt ppm H_2_O) and wet (20–100 wt ppm H_2_O) crystals exhibit comparable indentation hardness. Quartz yield strength, which is proportional to indentation hardness, seems to be unaffected by the intracrystalline H_2_O content when deformed under room temperature, high‐stress conditions. Pre‐indentation intracrystalline microstructure may have provided a high density of dislocation sources, influencing the first increments of low‐temperature plastic strains. Our results have implications for fault strength at the frictional‐viscous transition and during transient deformation by LTP, such as seismogenic loading and post‐seismic creep.

## Key Points

- Low‐temperature plasticity in quartz with varying intracrystalline H_2_O was investigated by spherical and Berkovich nanoindentation
- Naturally deformed, wet and dry quartz grains exhibit similar yield and post‐yield hardness during nanoindentation at room temperature
- Intracrystalline H_2_O content does not affect the strength of quartz in the low‐temperature plasticity regime

## 1 Introduction

Experimental rock deformation has demonstrated that the strength of quartz is significantly reduced by the presence of intracrystalline H_2_O either bonded to the crystal lattice or occurring as micro‐fluid inclusions, that is, quartz exhibits hydrolytic weakening (Ave Lallemant & Carter, 1971; Griggs, 1967; Griggs & Blacic, 1965; Tullis & Yund, 1980). Results from laboratory experiments established that hydrolytic weakening occurs in both synthetic and natural quartz crystals with intracrystalline H_2_O contents larger than 20–30 wt ppm (about 150 H/10^6^ Si; Stünitz et al., 2017 and references therein). Several different microphysical processes have been proposed to explain hydrolytic weakening in quartz: (a) hydrolyzation of Si‐O‐Si bonds around dislocations, consequently decreasing the resistance to dislocation motion (i.e., reducing the Peierls stress; Griggs, 1967), (b) enhanced dislocation generation around H_2_O clusters within the crystal lattice (McLaren et al., 1989; Stünitz et al., 2017), and (c) enhanced recovery through increased ionic diffusivities and faster dislocation climb (Post et al., 1996; Tullis & Yund, 1989).

Hydrolytic weakening has been experimentally observed in natural and synthetic quartz deformed at high homologous temperature, conditions for which dislocation climb and recovery processes control the overall strain rate (Fitz Gerald et al., 2006; Holyoke & Kronenberg, 2013; Kronenberg & Tullis, 1984; Mancktelow & Pennacchioni, 2004; Stipp et al., 2006; Tullis & Yund, 1980). However, there is considerable interest in the deformation of quartz at higher stresses and lower temperatures that are characteristic of the strength‐controlling portion of the continental crust near the frictional‐viscous transition (Goldsby et al., 2004; Lloyd, 2000; Stünitz et al., 2017; Trepmann et al., 2017). At such conditions, often referred to as the low‐temperature plasticity (LTP) regime, intracrystalline plasticity of quartz is controlled by the kinetics of dislocation glide, rather than recovery, and therefore any hydrolytic weakening is expected to come from either an effective decrease in Peierls stress (Griggs, 1967) or an increase in the dislocation nucleation rate (Stünitz et al., 2017). However, the possible effect of H_2_O, H^+^ or OH^−^ on the Peierls stress in quartz is still debated (Hartley & Wilshaw, 1973; McLaren et al., 1989; Trepied & Doukhan, 1982). Unfortunately, only very few experiments have been conducted at low temperatures, with conflicting results. For example, Griggs (1967) argued that hydrolytic weakening occurs in quartz, but only above a threshold temperature of ∼400°C. In contrast, Evans (1984) observed no hydrolytic weakening up to ∼900°C. In addition, both of these previous studies were conducted on synthetic quartz, making extrapolation to geological settings less clear. Thus, an improved understanding of the effect of intracrystalline H_2_O on the strength of natural quartz in the LTP regime is essential for future analysis of deformation at high stress in the crust.

Here we address the mechanical effects of intracrystalline H_2_O on the strength of quartz by analyzing the hardness of natural quartz grains with different H_2_O contents through a series of nanoindentation tests at room temperature. Nanoindentation is a valuable experimental procedure to investigate dislocation‐controlled plasticity at low temperature, given that the sample surrounding the indented volume experiences large self‐confining pressures that suppress brittle deformation (Evans & Goetze, 1979). We implement spherical and Berkovich nanoindentation tests to retrieve the hardness of individual quartz crystals with different amounts of intracrystalline H_2_O. Spherical nanoindentation provides an estimate of the yield hardness, which is proportional to the yield stress at which the material starts to deform plastically by dislocation glide (typically ∼2% indentation strain, Pathak & Kalidindi, 2015). The hardness retrieved from Berkovich nanoindentation is considered a proxy for the post‐yield resistance to plastic flow, representing the stress at constant indentation strain of 7% (Fischer‐Cripps, 2011). Nanoindentation has been integrated with measurements of intracrystalline H_2_O contents of the indented grains with secondary ion mass spectrometry (SIMS), and with electron backscatter diffraction (EBSD) analysis of the indented grains to identify the indented crystal orientation and the intracrystalline deformation microstructure.

## 2 Materials and Methods

### 2.1 Sample Material

The two investigated natural quartz aggregates occur in the granulite‐facies migmatitic gneisses of the Seiland Igneous Province (northern Norway), which were deformed during lower crustal shearing at *T* = 760–820°C and *P* = 0.75–0.95 GPa (Menegon et al., 2011). Rock chips (1 × 1 × 0.3 cm) were cut from the leucosome‐rich and from the leucosome‐poor domain of the migmatitic gneiss sample Ø15 analyzed in Menegon et al. (2011). The surface of the rock chip tested with nanoindentation is oriented perpendicular to the foliation and parallel to the stretching lineation. This surface was prepared for indentation and subsequent electron microscopy by iteratively polishing with diamond suspensions of decreasing grit size, finishing with a grit size of 0.05 μm.

**Figure 1:**
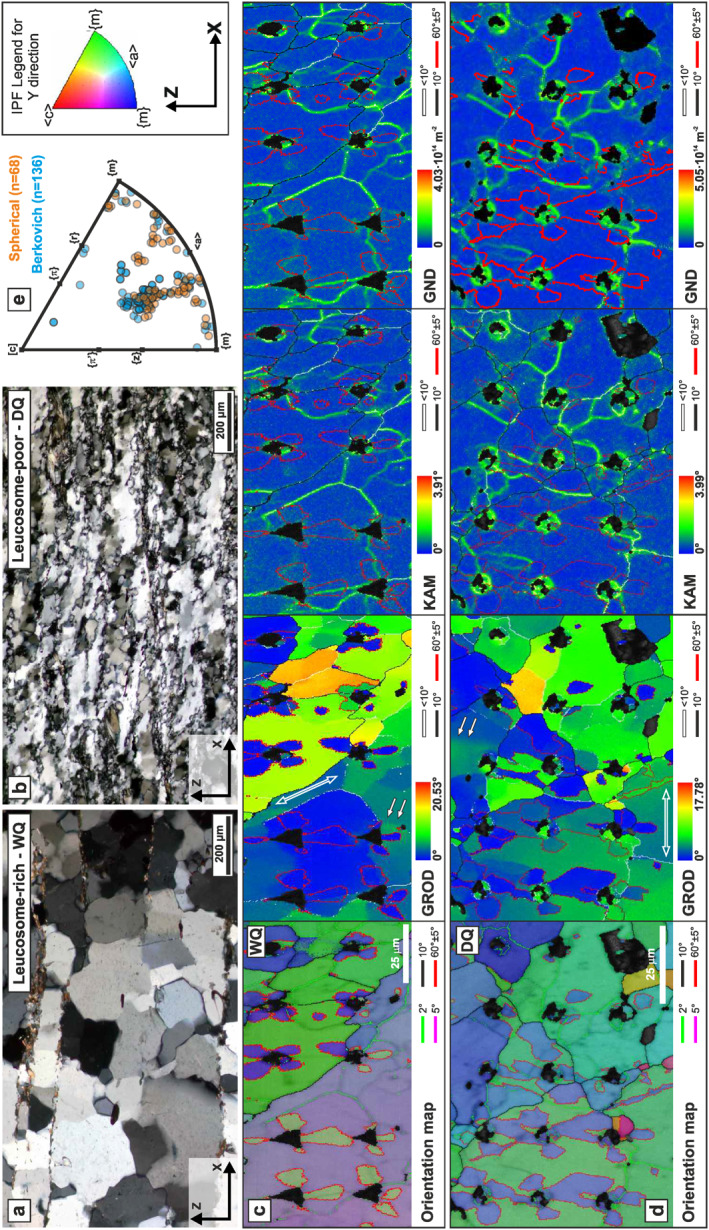
(a–b) Polarized light micrographs (crossed polarizers) of the microstructure of leucosome‐rich (wet quartz, WQ) and leucosome‐poor (dry quartz, DQ) quartz aggregates. (c–d) electron backscatter diffraction (EBSD) maps, for WQ and DQ respectively, including from left to right: orientation map color coded according to the IPF for the indentation direction (*Y*‐direction, key on upper right corner of the figure); grain‐reference‐orientation deviation (GROD) maps indicating the local misorientation of each pixel within a grain with respect to the average grain orientation; kernel‐average misorientation (KAM) maps indicating the local misorientation (up to 4°) at each pixel based on a 5 × 5 grid of neighboring pixels; map of the density of Geometrically Necessary Dislocations (GND) for dislocations exhibiting <a> as slip direction. Low‐misorientation angle boundaries are highlighted by green (>2°) and purple (>5°) lines in the orientation map, white lines (<10°) in GROD, KAM and GND maps. Black and red lines represent high‐misorientation angle boundaries, and Dauphiné twin boundaries, respectively. White, bold arrows point toward intracrystalline misorientation bands. White, hollow arrows are parallel to diffuse lattice curvature gradients. (e) IPF indicating the crystallographic directions of quartz crystals parallel to the indentation direction (Y) for Berkovich (light blue dots) and spherical (orange dots) nanoindentation as inferred from EBSD orientation maps.

Quartz grains exhibit internal deformation in both the leucosome‐rich and leucosome‐poor domains, as evidenced by undulatory extinction and the presence of subgrains (Figures 1a–1b). Quartz exhibits a similar c‐axis crystallographic preferred orientation (CPO) in both domains, with c‐axis maxima suggesting the activation of the basal <a> and rhomb <a> slip systems during dislocation creep (Menegon et al., 2011). Subgrains in leucosome‐poor domains are generally finer (<80 μm) than those observed in leucosome‐rich domains (100–200 μm). Further details on the initial sample microstructure can be found in Menegon et al. (2011).

### 2.2 Nanoindentation Tests and Data Analysis

Continuous Stiffness Measurement (CSM) nanoindentation tests were carried out at the Department of Materials, University of Oxford (UK), using an MTS Nanoindenter XP. Two series of nanoindentation tests were performed at room temperature (25°C) using Berkovich (three‐sided pyramid with half angle of 65.27°) and spherical (effective radius of 7 μm) diamond indenter tips. Tests were performed at constant indentation strain rate (defined as loading rate divided by load) of 0.05 s^−1^ up to a maximum depth of 2 μm and a maximum load of 530 mN. Stress‐strain curves for spherical nanoindentation tests have been computed from load‐displacement data following the approach of Pathak and Kalidindi (2015). The yield hardness has been obtained from graphical evaluation of stress‐strain curves (see Text S1 in Supporting Information S1 for details). Hardness and elastic modulus were computed from CSM Berkovich nanoindentation tests following the approach of Oliver and Pharr (1992). Of the total data set of nanoindentation tests (284 tests, 188 Berkovich + 96 spherical), only those effectively performed within single, inclusion‐free grains, away from grain boundaries, fluid inclusion traces, and macroscopic inter‐granular fractures (as inferred from EBSD and BSE images), have been considered for further mechanical analysis (136 Berkovich + 68 spherical). Further information about the experimental procedure and processing of mechanical data can be found in Text S1 in Supporting Information S1.

### 2.3 Secondary Ion Mass Spectrometry (SIMS)

Intracrystalline H_2_O content in quartz was measured with the Cameca IMS‐4f ion probe at the NERC Ion Microprobe Facility at the University of Edinburgh, UK. Measurements were acquired from the same optically clear quartz grains on which nanoindentation tests were performed, paying attention to avoid fluid inclusions, cracks, and grain boundaries (see Text S1 in Supporting Information S1 for details about sample preparation and analytical conditions).

### 2.4 Electron Backscatter Diffraction (EBSD)

EBSD analysis was performed on selected areas investigated by nanoindentation. EBSD analysis was performed at the Electron Microscopy Centre of the University of Plymouth (UK) using a JEOL 7001 FEG SEM equipped with a NordLys Max EBSD detector (AZtec acquisition and processing software, Oxford Instruments). EBSD results are presented as orientation maps and inverse pole figures (IPFs, Figures 1c–1e; S2). EBSD maps of the grain reference orientation deviation (GROD), and kernel average misorientation (KAM) were derived to evaluate the extent and distribution of intracrystalline deformation near each indent (Figures 1c and 1d; S2). Maps quantifying the distribution and density of Geometrically Necessary Dislocations (GND) with <a> as Burger vector were computed from KAM maps (Figures 1c and 1d; S2) using AZtec.

## 3 Results

### 3.1 SIMS—H_2_O‐Content Analysis

**Figure 2:**
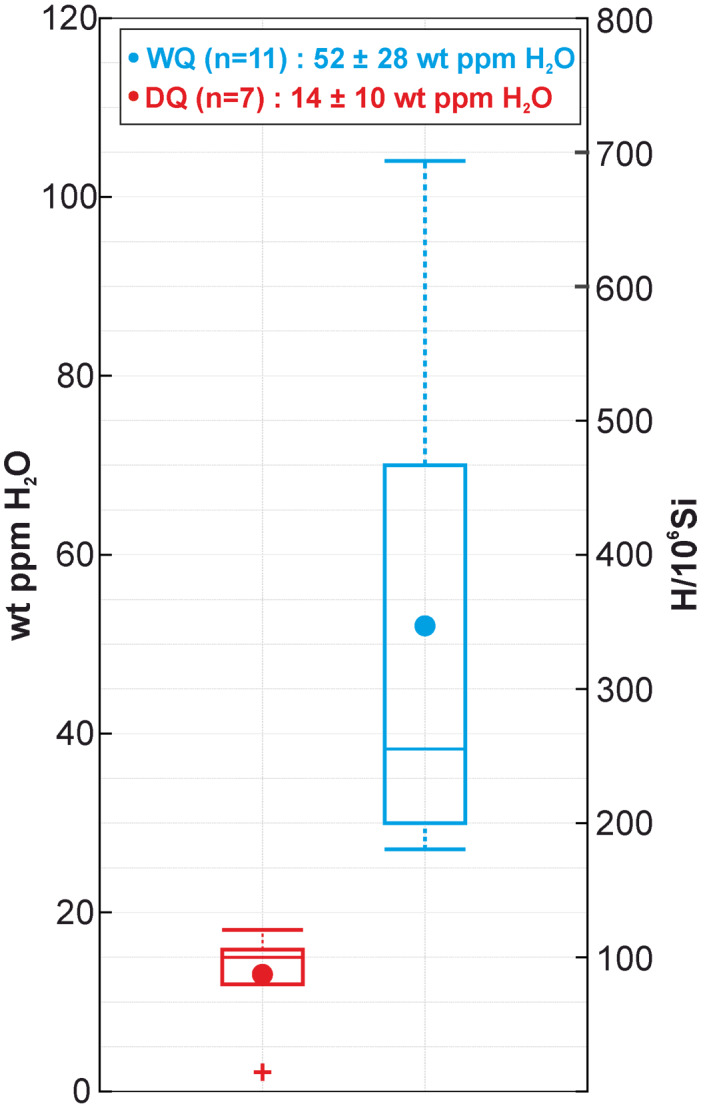
Box‐and‐whisker plot presenting the H_2_O content of dry quartz and wet quartz samples as obtained from secondary‐ion mass spectrometry analysis. The inset reports the mean and 2σ values of measured wt ppm H_2_O.

SIMS analyses from quartz in leucosome‐poor samples reveal H_2_O contents ranging between 15 and 27 wt ppm of H_2_O (average of 18 ± 10 [2σ] ppm over five measurements). Quartz in leucosome‐rich samples reveal H_2_O contents ranging between 2 and 104 ppm (average of 45 ± 56 [2σ] ppm over 13 measurements). Given the observed scatter in the measured H_2_O contents, SIMS data and the related results of nanoindentation tests and EBSD analyses have been divided into two datasets (“dry quartz”[DQ]; “wet quartz”[WQ]; Figure 2) considering 20 wt ppm H_2_O as the threshold above which quartz grains are commonly considered “wet” (Milke et al., 2013; Stünitz et al., 2017). Quartz grains with <20 wt ppm H_2_O have been assigned to the “DQ” data set (2–18 wt ppm H_2_O over seven measurements; Figure 2), whereas those with >20 wt ppm H_2_O to the “WQ” data set (27–104 wt ppm H_2_O over 11 measurements; Figure 2).

### 3.2 Nanoindentation Tests

#### 3.2.1 Spherical Nanoindentation Tests

**Figure 3:**
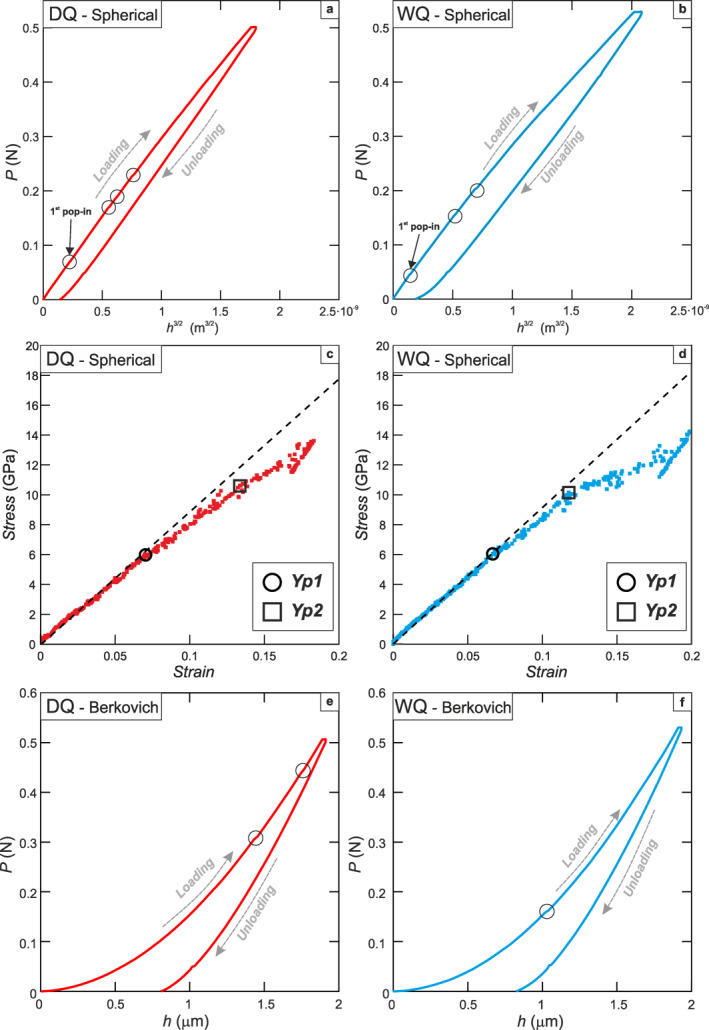
Load‐displacement and stress‐strain diagrams resulting from spherical and Berkovich nanoindentation tests. (a–b) Load‐displacement diagrams from spherical nanoindentation tests. Purely elastic behavior of the sample would be represented by a linear relationship between (Displacement)^3/2^ and Load. Circled points along load‐displacement curves represent the occurrence of pop‐ins during loading. (c–d) dry quartz and wet quartz stress‐strain curves from spherical nanoindentation. Both curves present two breakpoints (*Yp*_1_ and *Yp*_2_) at which the slope changes, and either could be taken to represent the yield conditions (see text for explanation). (e–f) Load‐displacement diagram of representative Berkovich nanoindentation tests.

**Figure 4:**
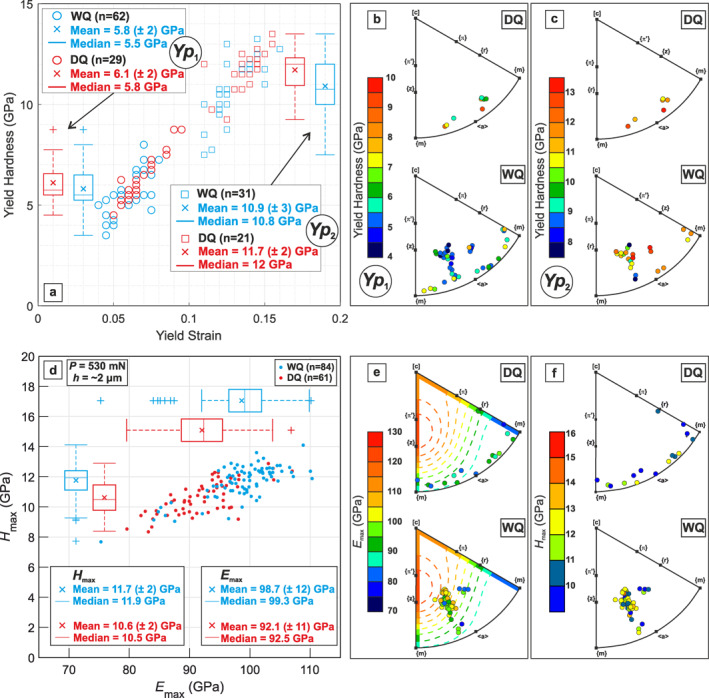
Results of hardness measurements from nanoindentation tests. (a) Stress‐strain scatter diagram and box‐and‐whiskers plot indicating the distribution of yield hardness for the identified breakpoints *Yp*_1_ and *Yp*_2_. (b–c) IPFs for quartz illustrate the yield hardness values for *Yp*_1_ and *Yp*_2_ for each tested crystallographic direction in DQ and WQ samples. (d) Hardness versus elastic modulus scatter plot indicating the distribution of *H*_max_ *−E*_max_ values at maximum load (530 mN) from Berkovich nanoindentation. The insets in (a) and (d) report the mean, median, and 2σ (in bracket) of the yield hardness, elastic modulus and hardness of each data set. (e–f) IPFs for quartz illustrating the elastic modulus and indentation hardness values, respectively, for each tested crystallographic direction. The dashed contour lines in (e) represent the expected variation of elastic modulus as calculated from the elastic tensor of dry and wet quartz at room temperature (Ogi et al., 2006).

Load‐displacement curves for spherical nanoindentation tests (Figures 3a and 3b) exhibit (a) residual displacements, indicating plastic deformation, and (b) rare and very small steps in the loading portion of the load‐displacement curve (“pop‐ins,” hollow circles in Figures 3a and 3b; Pathak & Kalidindi, 2015). The yield hardness is usually retrieved from graphic evaluation of stress‐strain curves as the breakpoint in the slope between the first segment of the curve, exhibiting a linear relationship between stress and strain, and the remaining part of the curve showing a generally less steep slope describing the plastic behavior of the sample (Pathak & Kalidindi, 2015). However, there can be ambiguity in stress‐strain curves, and in many of our tests it is possible to define two breakpoints (Figures 3c and 3d). To remove some subjectivity in picking yield points, for each indent we report the stress values of both points along the stress‐strain curve as *Yp*_1_ and *Yp*_2_. At stresses above both *Yp*_1_ and *Yp*_2_, the stress continues to increase with strain, which is consistent with the strain hardening previously observed in nanoindentation tests on other geological materials in the LTP regime (Kranjc et al., 2016; Kumamoto et al., 2017). The corresponding values of hardness at *Yp*_1_ and *Yp*_2_ are reported in Figure 4a for the whole data set. Both *Yp*_1_ and *Yp*_2_ occur over a wide range of stress conditions, yet are comparable for both WQ and DQ samples (Figure 4a). The hardness at *Yp*_1_ for WQ (5.8 ± 2 GPa [2σ]) is statistically indistinguishable from the value at *Yp*_1_ for DQ (6.1 ± 2 GPa). Similarly, hardness at *Yp*_2_ for WQ (10.9 ± 3 GPa) is also statistically indistinguishable from the value at *Yp*_2_ for DQ (11.7 ± 2 GPa). Some of the variability in yield hardness can be attributed to elastic anisotropy, as best evidenced by *Yp*_1_ in WQ, for which values of *Yp*_1_ derived from tests performed parallel to <a‐m> (6.0–8.5 GPa) are on average larger than those obtained from indentation parallel to <r‐z> (<6.0 GPa; Figure 4b).

#### 3.2.2 Berkovich Nanoindentation Tests

Load‐displacement curves from Berkovich nanoindentation tests exhibit a residual displacement of ∼0.8 μm (Figures 3e and 3f). Elastic moduli calculated at maximum load (530 mN) for WQ and DQ range between 84 and 107 GPa (98.7 ± 12 GPa), and 79 and 107 GPa (92.1 ± 11 GPa), respectively (Figure 4d). Elastic moduli are comparable (within 5%–10%) with the elastic modulus predicted for right‐handed α‐quartz (Ogi et al., 2006), with larger values for quartz samples indented parallel to <r‐z> than those obtained from samples indented parallel to <a‐m> (Figure 4e). Hardness values for WQ and DQ at maximum load conditions ranges between 9 and 13.5 GPa (11.7 ± 2 GPa), and 8 and 13 GPa (10.6 ± 2 GPa), respectively (Figure 4d). DQ samples indented parallel to <a‐m> directions exhibit significantly (up to 4 GPa) lower hardness values compared to WQ samples indented close to <r‐z> (Figure 4f). During sample loading, hardness decreases with increasing indentation depth into the sample (Figure S3 in Supporting Information S1).

### 3.3 EBSD Maps

Nanoindentation tests were mainly performed either parallel to directions halfway between <r> and <z> or normal to [c] (Figure 1e). Dauphiné twins are widespread around indents forming four‐fold lobes (Figures 1c and 1d), similar to observations in previous studies (Ferguson et al., 1987; Hartley & Wilshaw, 1973; Lloyd, 2000).

The EBSD maps highlight the similar pre‐indentation intracrystalline microstructures of the indented DQ and WQ grains. The KAM maps highlight the presence of low‐angle boundaries defining subgrains and characterized by relatively large lattice distortions (KAM in Figures 1c and 1d. Lattice distortion occurs as either localized misorientation bands or diffuse distortion gradients (white arrows in GROD maps, Figures 1c and 1d). Accordingly, the GND density maps computed from the KAM maps identify a heterogeneous density of <a> dislocations, with higher densities concentrated along low‐angle boundaries (Figures 1c and 1d). Additional EBSD maps and SE images of indents can be found in Figures S1–S2 in Supporting Information S1.

## 4 Discussion and Conclusion

The range of intracrystalline H_2_O content in DQ and WQ encompasses the transition from what is normally considered “dry” and “wet” quartz in deformation experiments, at around 20–30 wt ppm H_2_O (Stünitz et al., 2017).

The results of indentation experiments are notoriously difficult to compare directly to other types of mechanical tests because of the complicated deformation geometry and potential effects related to the scale of deformation (Kumamoto et al., 2017). For instance, the hardness in our Berkovich tests decreases with indentation depth, indicating the occurrence of a “size‐effect” in quartz (Thom et al., 2017; Figure S3 in Supporting Information S1). This effect is related to the effective decrease in dislocation density as the plastic zone under the indenter increases in volume (Pharr et al., 2010). However, we analyze our mechanical data at a consistent set of conditions such that size effects do not affect comparisons among datasets. Hardnesses derived from Berkovich indents were all measured at the same depth (2 μm), and yield hardnesses derived from spherical indents were all measured with a spherical indenter of the same radius. This consistency in testing conditions allows direct comparison between the WQ and DQ datasets.

Our comparison of indents conducted on wet and dry grains of quartz demonstrates that:

1. For the range of intracrystalline H_2_O content sampled here, the spherical yield hardness and the Berkovich hardness do not systematically differ between WQ and DQ grains. These observations suggest that neither the yield stress (and by proxy the Peierls stress) nor the post‐yield strength of quartz are affected by the intracrystalline H_2_O content in the LTP regime. Thus, this apparent lack of a reduction in Peierls stress suggests that hydrolytic weakening by covalent bond hydrolyzation is not efficient in the LTP regime. Similarly, recent results from nanoindentation tests on α‐quartz reported by Strozewski et al. (2021) have shown that indentation hardness of both synthetic and natural quartz crystals is independent from intracrystalline H_2_O even at higher temperatures (up to 500°C) and larger water contents.
2. Load‐displacement curves are characterized by rare, low‐intensity “pop‐ins,” which are usually inferred to represent the activation and multiplication of dislocations from dislocation sources, promoting plastic deformation. As the tip progressively indents the sample, the deformed volume increases proportionally, increasing the probability of activating dislocation sources and pre‐existing dislocations (Thom et al., 2017). Large intensity pop‐ins would suggest that large indentation volumes are necessary to activate dislocation motion and that, by inference, the densities of dislocations and dislocation sources in the starting material are relatively low. Conversely, as observed in our tests, the lack of substantial pop‐ins suggests a high density of dislocation sources is available (Kumamoto et al., 2017) within both WQ and DQ grains. We note that larger tips tend to produce smaller pop‐ins (Kumamoto et al., 2017), but the pop‐ins observed here are still exceptionally small (<10 nm) compared to those observed in other materials with even larger tips (10–100s of nm, Pathak & Kalidindi, 2015).
3. The lack of pop‐ins in both WQ and DQ indicates that the different intracrystalline H_2_O content did not influence the nucleation and multiplication of dislocations in our experiments (c. f. McLaren et al., 1989; Stünitz et al., 2017). Instead, we emphasize that WQ and DQ grains are characterized by the presence of low‐angle boundaries surrounding regions of high intracrystalline lattice distortion (Figures 1c and 1d), resulting from the geological, pre‐indentation crystal‐plastic deformation history (Menegon et al., 2011). Qualitatively, these substructures are indicative of an inherited high dislocation density of the natural samples (GND maps of Figures 1c and 1d). Therefore, the inherited high densities of dislocations and/or dislocation sources might have dominated over any possible influence of intracrystalline H_2_O on dislocation nucleation and multiplication. However, in nanoindentation experiments on olivine with and without pre‐existing dislocation structures, Kumamoto et al. (2017, see their Figure 2a) demonstrated that stress‐strain curves after pop‐ins (in material without pre‐existing structures) are identical to stress‐strain curves of material with pre‐existing structures. This observation suggests that only the first few percent of plastic deformation are influenced by the source density, and the rest of the stress‐strain curve is primarily controlled by the ease of dislocation glide. This concept is consistent with the analysis of Hobbs et al. (1972) describing the presence of an upper yield point and subsequent drop in stress in quartz single crystals. Thus, it is possible that a lack of initial dislocation content might lead to a difference in the initial yield between dry and wet quartz, but we suggest that any deformation beyond a few percent plastic strain would not be affected by water content.

Therefore, the kinetics of dislocation glide in the LTP regime will not be affected by the intracrystalline H_2_O. Similarly, recent results from complementary nanoindentation tests at higher temperatures (up to 500°C; Strozewski et al., 2021) suggest that dislocation glide kinetics may be insensitive to intracrystalline H_2_O even in strain‐free, H_2_O‐rich, synthetic crystals. Conversely, we speculate that the presence of abundant dislocation sources, inherited from geological deformation events, may play an important role in influencing the initial yield of natural quartz.

An important geophysical implication of our results is that the transient response of the middle and lower crust to short‐term seismogenic loading and post‐seismic creep by LTP (Trepmann et al., 2017; Wallis et al., 2021) will be independent of the intracrystalline H_2_O in the primary mineral phases. Hydrated portions of the crust will not be more prone to localize deformation associated with seismogenic loading and post‐seismic creep than anhydrous regions. Thus, the influence of water on short‐term mechanical behavior is significantly different from its influence on the long‐term strength and rheology of the middle and lower crust, which are notoriously affected by the availability of aqueous fluids (Bürgmann & Dresen, 2008; Jamtveit et al., 2019).

In addition, the observation that the first increments of plastic strain may be controlled by the availability of pre‐existing dislocation sources has relevant consequences for the strength of faults at the frictional‐viscous transition (FVT). The yield strength during the progressive ductile‐to‐brittle overprint of faults exhumed through the FVT (for example, low‐angle normal faults accommodating the exhumation of metamorphic core complexes: Axen, 2004) will be controlled by their pre‐existing microstructure rather than by the intracrystalline H_2_O. Furthermore, the yield strength of quartz during LTP preceding and controlling localization associated with microfracturing (Lloyd, 2000; Trepmann & Stöckhert, 2013) will not depend on H_2_O but rather on the availability of dislocation sources.

However, it is possible that increased densities of micro‐fluid inclusions (Stünitz et al., 2017) and/or intracrystalline H_2_O contents larger than those sampled here (i.e., >>100 wt ppm H_2_O) might outweigh the effect of pre‐existing microstructure in natural samples. Initial yield might be affected by water in strain‐free, H_2_O‐rich natural quartz crystals, for example, in magmatic quartz grains and in synkinematic quartz veins, which frequently localize strain at the base of the seismogenic zone in the middle crust (Ceccato et al., 2017; Marchesini et al., 2019; Pennacchioni et al., 2010). Further nanoindentation experiments on selected natural, unstrained quartz grains with different intracrystalline H_2_O are required to test this hypothesis. However, we suggest that the glide process controlling quartz LTP will not be affected by intracrystalline H_2_O content.

## Supporting information

Supporting Information S1

## Data Availability

Datasets available at http://dx.doi.org/10.17632/56sgvzvhvc.2.
