# Supplementary material for: Strength of Dry and Wet Quartz in the Low‐Temperature Plasticity Regime: Insights From Nanoindentation
Source: Geophys Res Lett. 2022 Jan 27;49(2):e2021GL094633. doi: 10.1029/2021GL094633 (PMC9286563; doi:10.1029/2021GL094633)
Supplement: Supplementary file 1 — Supporting Information S1 [file GRL-49-0-s001.docx]

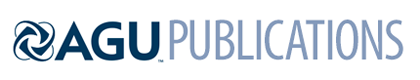


*Geophysical Research Letters*

Supporting Information for

**Strength of dry and wet quartz in the low-temperature plasticity regime: insights from nanoindentation**

Alberto Ceccato^1^, Luca Menegon^2,3^, Lars N. Hansen^4,5^

1 Dipartimento di Scienze Biologiche, Geologiche ed Ambientali – BiGeA, Università di Bologna – Alma Mater Studiorum, via Zamboni, 67, 40126 Bologna – Italy; 2 The Njord Centre, Department of Geosciences, University of Oslo, Postbox 1047 Blindern, 0316 Oslo, Norway; 3 School of Geography, Earth and Environmental Sciences, University of Plymouth, Drake Circus, Plymouth PL48AA, UK; 4 Department of Earth Sciences, University of Oxford, Oxford, UK; 5 Department of Earth and Environmental Sciences, University of Minnesota, Twin Cities, Minneapolis, MN, USA]

**Contents of this file**

Text S1

Figures S1 to S3

**Introduction**

The Supporting Information provided in this text files include additional specifics about the analytical techniques and data analyses presented in the main manuscript. Supplementary Figures are provided in order to integrated figures and diagrams displayed in the main manuscript.

Text S1.

*Ø15 Sample additional information from Menegon et al. (2011).* FTIR measurements reveal that quartz in leucosome-rich domains, within which H_2_O preferentially partitioned, has an average H_2_O content of 46 wt ppm H_2_O with most measurements > 40 wt ppm H_2_O. In contrast, quartz in leucosome-poor, H_2_O depleted, domains, has low intracrystalline H_2_O contents (generally < 30 wt ppm H_2_O, and never > 62 wt ppm H_2_O; Menegon et al., 2011).

*Nanoindentation experiment procedure and mechanical-data processing.* The tip calibration function has been retrieved from nanoindentation tests performed on a standard of known hardness and modulus (fused silica). An area-versus-depth function was defined for the Berkovich tip and the effective tip radius was defined for the spherical tip. Tests were performed at constant indentation strain rate (load divided by the loading rate) of 0.05 s^-1^ up to a maximum depth of 2 µm and a maximum load of 530 mN. The maximum load was maintained constant for 10 s. During unloading, the indenter tip was held fixed at 55 mN to quantify the effects of thermal drift on the finite residual displacement after indentation. Based on this measured drift rate, a correction was applied to the displacements throughout the loading curve.

Load-displacement data from continuous stiffness measurements (CSM) made during Berkovich nanoindentation tests have been analysed following the standard approach for CSM nanoindentation described in Oliver and Pharr (2004), providing the elastic modulus, *E,* and indentation hardness, *H,* of the sample as a function of indentation depth. The computation of elastic modulus during CSM indentation tests is based on the ratio between contact stiffness and contact area (Olivier and Pharr, 2004),

$E=\frac{\sqrt{\pi}}{2}\frac{S}{\sqrt{A}}$,

where *E* is the sample elastic modulus, *S* is the measured contact stiffness, and *A* is the contact area of the indenter. The indentation hardness *H* is calculated from the ratio between the imposed load and contact area,

$$H=\frac{P}{A}$$

where, *P* is the load and *A* is the contact area.

Load-displacement data from spherical nanoindentation tests have been analysed following the approach described in Oliver & Pharr (1992) and Pathak & Kalidindi (2015) to retrieve indentation stress-strain curves for each experiment. Raw load-displacement data have been corrected subtracting from the measured displacement, *h*, and load, *P*, respectively, the effective zero-point of initial contact and the corresponding zero-point load to obtain the corrected load, *P_corr_*, and the corrected displacement, *h_corr_*. The corrected load, *P*_corr_, is then plotted against the displacement *h*_corr_*^3/2^* to highlight the deviation from purely elastic, Hertzian behaviour, which is linear in that construction (Pathak & Kalidindi, 2015). Indentation stress, σ, during loading was calculated as

$\sigma=\frac{P_{\mathrm{corr}}}{\pi}\frac{1}{a^{2}}$,

where *a* represent the contact radius determined from the ratio between measured contact stiffness, *S,* and sample elastic modulus, *E*_ind_*_,_*,

$a=\frac{S}{2E_{\mathrm{ind}}}$.

Indentation strain, ε, was calculated as

$\varepsilon=\frac{4h_{\mathrm{corr}}}{3\pi a}$.

The identification of the yield point is made through the optical evaluation of the stress-strain curve computed from load-displacement data from spherical nanoindentation. The yield point is identified as the main breakpoint in the slope of the stress-strain curve, which is characterized by a linear segment during the first loading increments of the test followed by another segment characterized by a different slope. This operation can be rather subjective when the stress-strain curve presents subtle changes in the slope.

*Electron Backscatter Diffraction (EBSD) additional information*. Samples have been carbon-coated without any additional (neither mechanical nor chemical) polishing of the surface to preserve residual indentations (Fig. S1). EBSD maps were acquired with a step size of 0.3 µm using a 20 kV acceleration voltage, a working distance between 17 and 20 mm, and processed with the AZtec software package (Oxford Instruments).

*Secondary ion mass spectrometry (SIMS) additional information.* Prior to analysis and gold coating, the samples were placed in an oven at 105°C for 30 minutes to remove surface humidity. The samples were coated with a thin gold film (<0.02 µm) and kept in the SIMS chamber under high vacuum conditions (5 x 10-9 Torr) for >48 hours prior to analysis. Analysis was performed with a 5 nA primary beam of 16O- ions accelerated to 14.5 kV. Basaltic glass standard St81A9 (Lesne et al., 2011) was used to calibrate the H2O contents, whereas an anhydrous olivine standard (Kilbourne Hole) was used to correct for background 1H signals. H2O contents calculated as wt ppm H2O have been converted to H/106 Si applying the conversion of Gleason and DeSisto (2008) (1 wt ppm H2O = 6.67 H/106 Si). To reduce the risk of surface contamination, the first few nm of surface material were removed by sputtering the spot for 4 minutes while rastering the beam over a 25 μm grid before each measurement. Measurements were acquired using a beam spot size of 20 µm (±5 µm). Each analysis consisted of 20 repeats (cycles) of the isotopes ^1^H, ^27^Al, ^30^Si, and ^49^Ti. For hydrogen, only the last 10 or 15 cycles were averaged, as ^1^H signals usually dropped during the first cycles before stabilizing. Basaltic glass standard St81A9 (Lesne et al., 2011) was used to calibrate the H_2_O contents, whereas an anhydrous olivine standard (Kilbourne Hole) was used to correct for background ^1^H signals. The measured background signal ranged from 4 to 12 ppm H_2_O. Water contents calculated as wt ppm H_2_O have been also converted to H/10^6^Si applying the conversion of Gleason & DeSisto (2008) (1 wt ppm H_2_O = 6.67 H/10^6^Si).


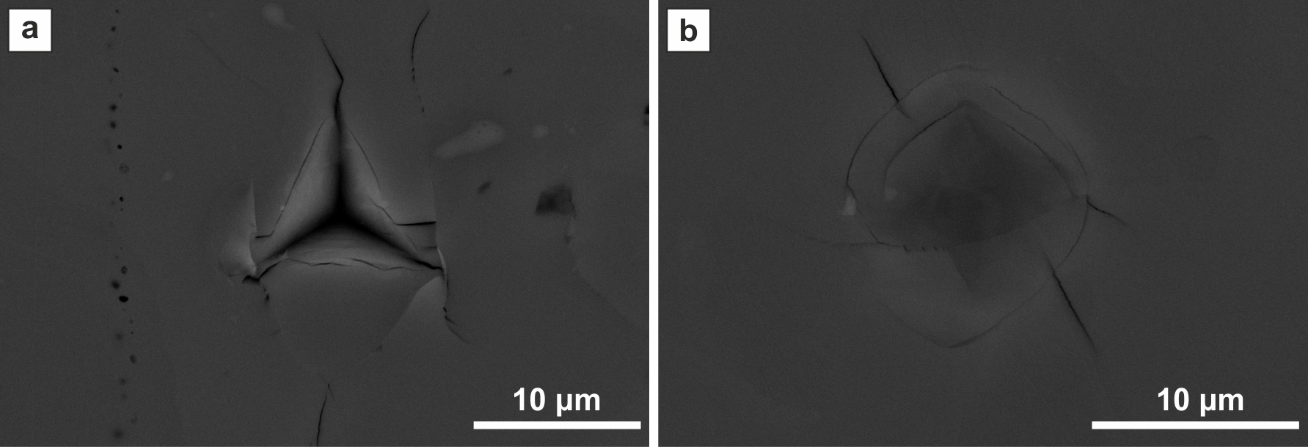


Figure S1. Secondary-electron SEM image of the indenter spots of Berkovich (a) and spherical (b) nanoindentation test. Berkovich and spherical nanoindentations exhibit characteristic morphological features including (i) pile-up of material around the indent and (ii) radial, median, and ring cracks around the indent.


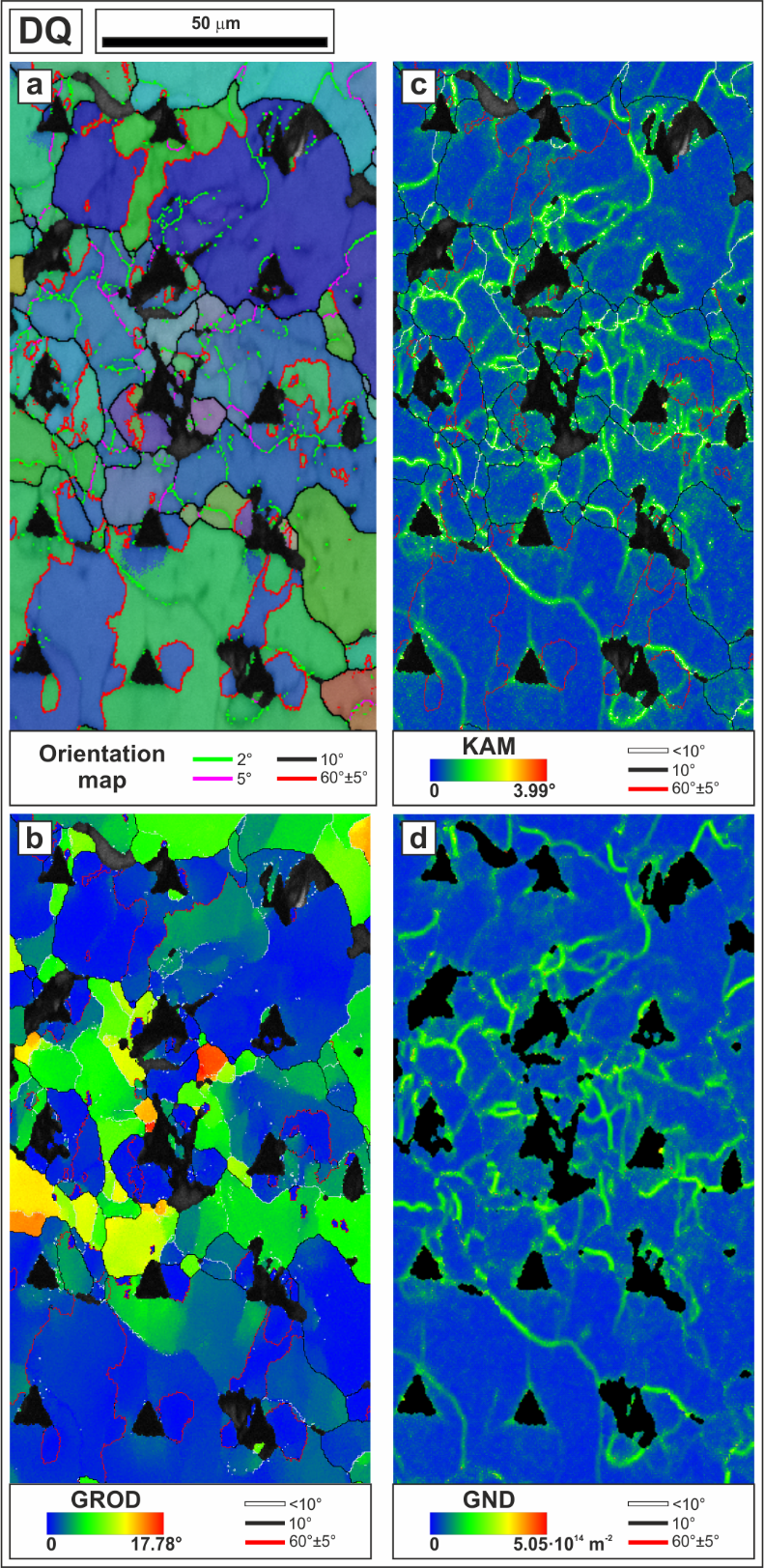


Figure S2. Supplementary EBSD mapping results on a DQ sample indicating the area selected for Berkovich nanoindetation tests. (a) Orientation map colour coded according to the inverse pole figure for the Y-direction (see Fig. 1 of the main text), which corresponds to the indentation direction; (b) Grain Reference Orientation Deviation (GROD) maps showing the local misorientation at each pixel within a grain with respect to the average grain orientation; (c) kernel average misorientation (KAM) maps showing the local small misorientation (up to 4°) at each pixel with respect to a 5x5 grid of neighboring pixels; (d) Map representing the density of Geometrically Necessary Dislocation for dislocation exhibiting <a> as slip direction, as computed from KAM maps in AZtec.


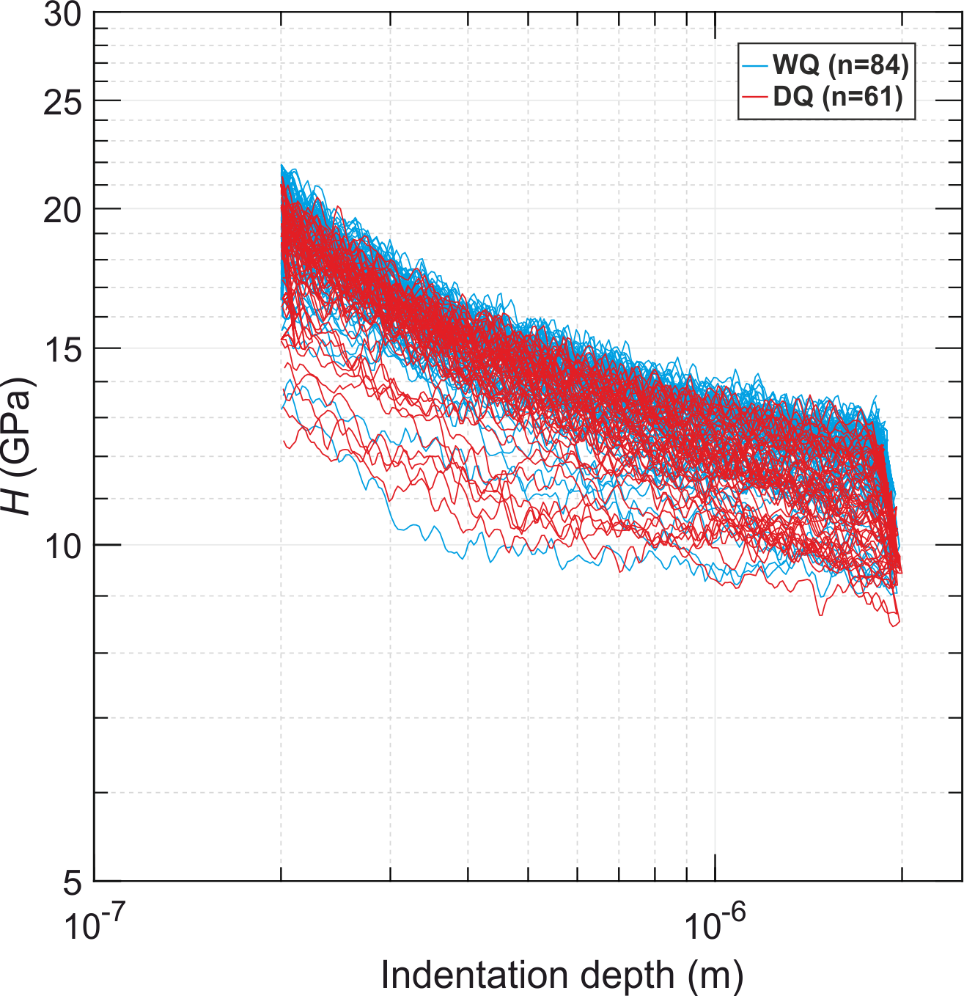


Figure S3. Variation of hardness H with indentation depth as retrieved from Berkovich nanoindentation tests.
